# Researches on a Disease Yet but Little Known, Which Has Received the Name of Softening of the Brain

**Published:** 1820-12

**Authors:** 


### CRITICAL ANALYSIS

OF

### RECENT PUBLICATIONS, IN THE DIFFERENT BRANCHES OF MEDICINE AND SURGERY;

### SELECT MEMOIRS, AND HISTORIES OF CASES;

### In the Literature of Foreign Nations.

Πατρίδες ἄρα
Ανδράσιν, ὦ πατραῖς ἄνδρες, ἀγαλλόμεθα.

*Recherches sur une Maladie encore peu connue qui a reçu le Nom de Ramollissement du Cerveau.* Par L. Rostan.—i. e. *Researches on a Disease yet but little known, which has received the Name of Softening of the Brain.* (Published in the *Nouveau Journal de Médecine*, September 1820.)

We noticed this morbid state of the brain in a former Number of this Journal, (Jan. 1819,) and gave then an abstract of such observations as had been made respecting it: but, though many instances of it have been related by different authors, as they witnessed it in an unexpected manner in their pathological researches, no one before Mr. Rostan made it an express object of their study, and assumed it as the *ex professo* subject of a treatise. In the present memoir, he considers the history of the disease in a general manner, in some preliminary remarks, and adduces the examples of it which have occurred to his own observation: he proposes to treat, successively, in subsequent memoirs, 1°, of the disease in its state of complication; 2°, of the duration of the disease; 3°, of its frequency; 4°, of the morbid changes with which it is accompanied; 5°, of the nature of the disease; 6°, of its termination, and of the prognosis of it; 7°, of its causes; 8°, of its treatment; 9°, of its diagnostics. We shall now give an abstract of the present memoir.

It is about nine years since Mr. Rostan first remarked this species of disease, at the Salpetrière: in that period he has witnessed several instances, and he relates eight cases in detail. On discussing, in the first instance, the proper appellation for this affection, he objects to the term "*chronic inflammation of the brain,*" which has been proposed; since it is often, he says, far from being a chronic disease, and he is not convinced that it is always of an inflammatory nature, though he thinks that it may in some cases merit the term *encephalitis.* He contents himself with giving to it an appellation which is descriptive of the most striking appearance it presents, a *softening of the brain.*

There are, he says, two distinct and well-marked periods in the disease.

The first presents only some vague phenomena which happen in numerous other affections, and which in themselves are of hardly any value as signs, though they become of the highest importance, and serve considerably to characterize the malady, when it is manifested by the symptoms of the second period. They are, indeed, of so much importance, that, when they have not existed, or when they have not been observed, the diagnosis is extremely obscure. The following are the precursory phenomena above alluded to. The patient is afflicted with a fixed pain in the head, which is obstinate and intolerable. This pain ordinarily resists all the means employed for its alleviation: it continues for a greater or less length of time, which may be several days or several months. This pain is not a constant precursor of the disease. Vertigo renders the step of the patient vacillating; the intellectual faculties become dull; the perceptions are slow; the judgment is not exerted without much difficulty; the memory is weak and treacherous; the imagination is extinct; the ideas are confused: the answers of the patient are, however, proper; but they are given in a tardy manner, and only after very long consideration. The tongue may be embarrassed in its movements, but sometimes the patient expresses himself with remarkable rapidity; his disposition of mind changes, he becomes morose, taciturne, peevish, and sometimes indifferent to external objects. There is often much tendency to sleep. The patient does not become delirious, but the persons who are about him perceive that his intelligence is not quite in its natural state. Besides those symptoms, creeping sensations, and often numbness, are experienced in one of the limbs, ordinarily towards the fingers; there is difficulty in seizing objects, or else a stiffness of the limb very often approaching to a permanent contraction of it: its sensibility is not always diminished in proportion to its contractility; sometimes, indeed, it happens that the sensibility is so much increased that the slightest touch causes the patient to cry out. These pains cannot be confounded with rheumatism, as there is neither heat, redness, nor tumefaction,—phenomena which always accompany rheumatism when it is acute. Sometimes there is delirium, with extreme agitation and febrile symptoms; and, lastly, mental alienation, senile demency, often accompany this softening of the brain, as Mr. Georget, as well as Mr. Rostan, have ascertained.

Intolerance of light, and strabismus, have but rarely been observed by Mr. Rostan to precede the lesion under consideration; but a dimness of sight, and even blindness, are not unfrequently precursory phenomena. There is often ringing noise in the ears; occasionally the slightest noise is insupportable, but more frequently the acuteness of the sense of hearing is diminished. The smell and taste are but rarely so deranged as to lead the patient to complain of them. Many of the phenomena just enumerated, the author acknowledges, are common to this disease and ordinary apoplexy, but he states that he shall establish the distinction and the value of each symptom, when he treats of the diagnosis.

Such are the precursory affections of relative or animal life: the functions of organic life are also often deranged during this period. The appetite is diminished, the thirst increased; digestion is difficult; the mouth is clammy, and the tongue white. Nausea, and even vomiting of an abundance of green, porraceous, bilious matter, often occur. The epigastrium, as well as the rest of the abdomen, is very sensible to pressure. Diarrhœa is sometimes present, but more frequently there is constipation, or rather a tardiness in the evacuation of the rectum. The stools are but rarely passed involuntary at this period: it is not so with the evacuation of the urine, which the patient, for the most part, has much difficulty in retaining. The quantity of the latter secretion is often less than natural. Respiration is but very rarely disturbed, although the patient sometimes complains of a slight sense of suffocation, or of oppression of breathing; it is more frequently inordinately slow than quickened, though difficulty of breathing is ordinarily manifested with acceleration of it. The pulse is very variable; it is but rarely increased in frequency; it is sometimes inordinately full, and in certain cases it is slower than natural. The symptoms presented by the other functions, as those of *absorption, exhalation,* the *secretions* and *excretions*, and *nutrition*, furnish no constant phenomena, nor such as are worthy of being noticed.

It is not rare for some intense internal phlegmasia, thoracic or abdominal, to precede the softening of the brain. Mr. Rostan has seen it, in some cases, preceded by a general inflammatory diathesis; all the viscera were inflamed, the lungs were hepatized, the pleura covered with false membranes, and the digestive tube inflamed throughout its whole extent. These last examples are but rare. It is more common to see the symptoms of the second period of the disease show themselves in a person who has been affected only with violent enteritis, or an intense peripneumony.

In the *second period,* the patient, after having presented some of the signs above enumerated, either gradually in a more or less rapid manner, or suddenly, loses the use of one of his limbs, sometimes of one-half of his body. If he is standing, he falls. It even happens, when he is lying down, that he falls from his bed on the floor. Most frequently he does not lose the use of his senses, and preserves his intelligence; but he has extreme difficulty in replying to the questions put to him, and it is often only by gesticulations that he expresses himself. There are cases in which the coma is perfect, but these cases are rare. When the comatose state has come on suddenly, as well as the paralysis, the patient commonly regains his consciousness on the day after the attack, and the inexperienced physician does not fail to felicitate himself on the success of his treatment; but new accidents soon take place to dissipate his illusion. The symptoms are again aggravated; intelligence and the sensual functions are entirely abolished; the patient falls into a state of complete coma; his limbs become immovable; and he dies at the end of a few days, ordinarily between the fourth and the fifteenth, presenting in the greater number of cases the symptoms of typhous fever.

The state of the limbs, in the course of this period of the disease, is not the same in all patients. The most frequent is that of diminution or abolition of the power of motion. The patient frequently experiences a sense of numbness, great heaviness, creeping or stinging sensations, and at last intolerable and shooting pains, increased on the limb being touched. It is not very rare to observe a great degree of stiffness, or an invincible contraction, of the affected sido. The forearm is bent on the humerus; there is much difficulty in bringing the limb into the straight position, and this is then preserved only for a moment. A much more rare state of the limbs, is that in which they are affected with convulsions.

The face may be either pale or strongly injected with blood, according to different circumstances. The pain in the head, which existed before the manifestation of the symptoms of the second period, now increases in intensity: this symptom even comes on when the patient had not previously suffered it. If he be asked where he feels uneasiness, sometimes after the first, but more frequently after the second or third, time of being thus questioned, he carries the arm, of which he preserves the power of motion, to some part of his head. It is remarkable that this is, almost always, precisely on the seat of the disease, and on the side opposite to that which is paralytic. When the patient has been delirious, the delirium persists after the occurrence of the palsy, but it is now more taciturn. Vomiting, first of food, then of bile, is often observed. The belly often furnishes evidence of extreme sensibility. Sometimes the feces are passed involuntarily, but more frequently there is constipation of the bowels. The urine generally escapes unknown to the patient; the respiration is often oppressed. The pulse is sometimes more frequent, and stronger, than ordinary.

Mr. Rostan has seen but one case in which the symptoms diminished in severity in the second period of the disease, and in this instance they soon returned with their former severity. The progress of the disease is, then, essentially continuous, and it is always increasing in violence. There are, besides, evening paroxysms, when inflammation of any organ is present.

A series of cases, constituting the sources whence the general observations above designated have been derived, is related by Mr. Rostan. It would be useless to select and transcribe here any one in particular, as the phenomena of it would only serve to show the foundation of some part of the author's general views. The appearances of this morbid state of the brain, in its ordinary form, were described in this Journal on a former occasion, (Jan. 1819;) and the account given of them by Mr. Rostan does not present any remarkable peculiarities or deviations from that to which we allude.

The present memoir concludes with some observations respecting the anomalies of this affection in its simple state. "These," he says, "involve the diagnosis of the disease with much uncertainty, and may even render it impossible, not from want of accuracy of observation, but from the nature of the symptoms."—"Some cases of this affection present no precursory symptoms, others evince such as are entirely contrary to the ordinary form of the disease, and some present symptoms insufficient to characterise it." The difficulties, he adds, are sometimes increased by the patient, although apparently possessing all his intelligence, having lost the remembrance of his previous state; and it is only from the persons who have been about him that we can then obtain the necessary important information.

